# SARS-CoV-2 infection induces a long-lived pro-inflammatory transcriptional profile

**DOI:** 10.1186/s13073-023-01227-x

**Published:** 2023-09-12

**Authors:** Jia-Yuan Zhang, Justin P. Whalley, Julian C. Knight, Linda S. Wicker, John A. Todd, Ricardo C. Ferreira

**Affiliations:** 1 JDRF/Wellcome Diabetes and Inflammation Laboratory, Wellcome Centre for Human Genetics, Nuffield Department of Medicine, NIHR Oxford Biomedical Research Centre, University of Oxford, Oxford, UK; 2 Wellcome Centre for Human Genetics, Nuffield Department of Medicine, University of Oxford, Oxford, UK; 3 Center for Cancer Cell Biology, Immunology and Infection, Chicago Medical School, Rosalind Franklin University of Medicine and Science, North Chicago, IL USA; 4 Chinese Academy of Medical Sciences Oxford Institute, University of Oxford, Oxford, UK

**Keywords:** COVID-19, SARS-CoV-2, Post-acute sequelae of COVID-19 (PASC), IL-2-induced anti-inflammatory signature, NF-kB, Single-cell multiomics

## Abstract

**Background:**

The immune response to severe acute respiratory syndrome coronavirus 2 (SARS-CoV-2) infection in COVID-19 patients has been extensively investigated. However, much less is known about the long-term effects of infection in patients and how it could affect the immune system and its capacity to respond to future perturbations.

**Methods:**

Using a targeted single-cell multiomics approach, we have recently identified a prolonged anti-inflammatory gene expression signature in T and NK cells in type 1 diabetes patients treated with low-dose IL-2. Here, we investigated the dynamics of this signature in three independent cohorts of COVID-19 patients: (i) the Oxford COVID-19 Multi-omics Blood Atlas (COMBAT) dataset, a cross-sectional cohort including 77 COVID-19 patients and ten healthy donors; (ii) the INCOV dataset, consisting of 525 samples taken from 209 COVID-19 patients during and after infection; and (iii) a longitudinal dataset consisting of 269 whole-blood samples taken from 139 COVID-19 patients followed for a period of up to 7 months after the onset of symptoms using a bulk transcriptomic approach.

**Results:**

We discovered that SARS-CoV-2 infection leads to a prolonged alteration of the gene expression profile of circulating T, B and NK cells and monocytes. Some of the genes affected were the same as those present in the IL-2-induced anti-inflammatory gene expression signature but were regulated in the opposite direction, implying a pro-inflammatory status. The altered transcriptional profile was detected in COVID-19 patients for at least 2 months after the onset of the disease symptoms but was not observed in response to influenza infection or sepsis. Gene network analysis suggested a central role for the transcriptional factor NF-κB in the regulation of the observed transcriptional alterations.

**Conclusions:**

SARS-CoV-2 infection causes a prolonged increase in the pro-inflammatory transcriptional status that could predispose post-acute patients to the development of long-term health consequences, including autoimmune disease, reactivation of other viruses and disruption of the host immune system-microbiome ecosystem.

**Supplementary Information:**

The online version contains supplementary material available at 10.1186/s13073-023-01227-x.

## Background

The recent COVID-19 pandemic has led to unprecedented collaborative efforts to elucidate the mechanisms of the immune response to SARS-CoV-2 infection and the development of clinical symptoms in COVID-19 patients. Although we now have a good understanding of the initial phases of the disease, less is known about the long-term effects of infection, associated with the development of post-acute sequelae of COVID-19 (PASC) [1]. The persistence of elevated infection rates and the risk of further outbreaks caused by novel variants make this a critical healthcare concern, particularly considering the increased risk of PASC posed by reinfection [2]. Over time, the breadth of studies dedicated to understanding COVID-19 have led to a rapid release of large publicly available single-cell transcriptomics datasets profiling the immune response to viral infection and disease outcome, in both the acute and post-acute phases of the disease, enabling detailed investigation of the long-term effects of COVID-19.

Recently, we have employed targeted single-cell multiomics to characterise the immune response to low-dose IL-2 immunotherapy in newly diagnosed type 1 diabetes patients from the DILfrequency study [3]. We identified a prolonged anti-inflammatory gene expression signature induced by low-dose IL-2 treatment (IL2-AIS) in all the T and NK cell subsets studied [4]. The transcriptional changes associated with the IL2-AIS detected in samples taken 28 days after the last IL-2 injection were not anticipated since Treg frequencies had returned to baseline by this timepoint. The known functions of many of the genes in the signature indicated a shift towards an anti-inflammatory lymphoid environment, including the upregulation of the negative regulator of cytokine signalling *CISH* and the modulation of a suite of tumour necrosis factor (TNF)-inducible genes.

Previous reports have associated differential expression of several constituent genes of the IL2-AIS, including *CISH*, *AREG*, *DUSP2*, *NFKBIA* and *TNFAIP3*, in COVID-19 patients [5, 6]. Nevertheless, the exact scale and dynamics of these changes remain unclear. Here, based on published single-cell transcriptomic data from two large COVID-19 cohorts, the Oxford COVID-19 Multi-omics Blood Atlas (COMBAT) [5] and the INCOV [6] cohorts, we show that a core set of co-regulated genes from the IL2-AIS is modulated in the opposite direction in blood T, B and NK cells as well as in monocytes of COVID-19 patients. A third whole-blood transcriptomic dataset from a longitudinal cohort of convalescent COVID-19 patients [7] provides additional evidence supporting the longevity of these transcriptional alterations in blood. This pro-inflammatory signature is progressively induced after the onset of clinical symptoms and is maintained for at least 2 months. Gene pathway analysis suggests that the transcription factor NF-κB could have a central role in the regulation of the observed transcriptional alterations, suggesting the prolonged activation of classical pro-inflammatory cytokine signalling pathways following viral infection. Our data shed new light into the long-term effects of SARS-CoV-2 infection on the immune system that could provide a mechanistic link for the clinical sequelae that persist in a subset of COVID-19 patients.

## Methods

### Single-cell RNA-seq datasets

The DILfrequency dataset [8] contains single-cell sequencing data of 39 samples from 13 adult participants receiving low-dose IL-2 immunotherapy. For each participant, three longitudinal samples were taken on day 0 (before the first IL-2 injection), day 27 (before the last IL-2 injection) and day 55 (4 weeks after the last IL-2 injection). Each sample contained five major cell types — CD4^+^ regulatory T cells (Treg), CD4^+^ conventional T cells (Tconv), CD8^+^ T cells, CD56^bright^ NK cells and CD56^dim^ NK cells — isolated by fluorescence-activated cell sorting (FACS) from peripheral blood mononuclear cells (PBMCs) and labelled with antibody-derived tags. Only day 0 and day 55 samples were analysed in the current study, with one sample (day 55 sample from participant P8) excluded due to quality control reasons previously described [4]. The DILfrequency dataset also contained cells stimulated with phorbol myristate acetate (PMA)/ionomycin, which were excluded from the current study.

The COMBAT dataset [9] contains single-cell sequencing data from PBMC isolated from 64 hospitalised COVID-19 patients, 13 non-hospitalised (community) COVID-19 patients, 12 critically ill influenza patients recruited from the intensive care unit (ICU), 23 hospitalised patients with all-cause sepsis and ten healthy control participants. PBMCs were classified into different cell types based on their gene expression clusters, protein markers and B- and T-cell receptor sequencing data. Pseudo-bulk samples with less than 2000 total transcript counts or less than 500 IL2-AIS transcript counts were excluded. This resulted in the exclusion of one critical COVID-19 patient and one community COVID-19 patient.

The INCOV dataset [10] contains single-cell sequencing data of 451 samples from 178 COVID-19 patients, with up to three longitudinal samples taken from each patient. Each sample contained PBMCs, with cell type information annotated bioinformatically based on clustering results. Twenty-four samples from ten immunocompromised or immunosuppressed patients were excluded from the current study.

The baseline characteristics of the participants in each study are summarised in Additional file 1: Table S1. The total mRNA counts in each pseudo-bulk sample are summarised in Additional file 2: Table S2.

### Generation and normalisation of pseudo-bulk expression data

Pseudo-bulk expression matrices were generated for each dataset by aggregating mRNA counts from cells from the same donor, time point (if applicable) and major cell type. For the DILfrequency dataset, which was based on a custom mRNA panel of 585 transcripts, pseudo-bulk samples with less than 100 total mRNA counts were removed. For the COMBAT and INCOV datasets, which were based on whole-transcriptome sequencing, pseudo-bulk samples with less than 2000 total mRNA counts were removed. The pseudo-bulk expression matrices for each dataset were normalised by dividing raw counts with sample-specific scale factors calculated using the median-of-ratios method previously described [11].

### Differential expression analyses

Differential expression analyses were performed separately for each dataset based on the pseudo-bulk expression matrix using DESeq2 [12]. For the DILfrequency dataset, the likelihood ratio test was used, with a full model including time points and the participants as independent variables, and a reduced model including only participants as the independent variable. For the COMBAT dataset, the Wald test was used, with patient groups (COVID-19 or healthy control) as the independent variable. For the INCOV dataset, considering the samples were taken during a wide range of time post COVID-19 symptoms, for each participant, we assigned the earliest sample taken 0–14 days post COVID-19 symptoms as the acute phase sample, and the earliest sample taken 29–84 days post COVID-19 symptoms as the post-acute phase sample. Only participants that have both acute and post-acute phase samples available were included in the differential expression analysis. The cutoff time points for acute and post-acute phase samples were selected to maximise the number of available participants, while minimising the heterogeneity within each group. The likelihood ratio test was used, with a full model including sampling time points (acute or post-acute phase) and participants as independent variables, and a reduced model including only participants as the independent variable. For all datasets, the apeglm method [13] was applied to shrink the resulting fold change values, and the Benjamini–Hochberg FDR correction was applied after pooling all resulting *P* values.

### Deriving the IL-2 induced anti-inflammatory signature score

The *Day 55 signature* induced by low-dose IL-2 treatment reported previously for the DILfrequency study [4] is referred to here as “Anti-Inflammatory Signature induced by IL-2 treatment (IL2-AIS)”. For the DILfrequency dataset, the IL2-AIS scores were calculated as previously described based on the normalised pseudo-bulk expression levels of the 20 upregulated signature genes (*CISH*, *TNFSF14*, *OAS1*, *GIMAP7*, *GIMAP5*, *TNFSF10*, *TAGAP*, *STAT1*, *MYC*, *FASLG*, *CX3CR1*, *PTGDR2*, *CRTAM*, *EOMES*, *IL32*, *CCR10*, *CCR1*, *CXCR1*, *CD40LG* and *ID3*) and the 21 downregulated signature genes (*AREG*, *DUSP5*, *TNFAIP3*, *RGS1*, *CXCR4*, *DUSP2*, *DUSP4*, *DDIT4*, *NFKBIA*, *FOSL2*, *NFKBIZ*, *ZBTB16*, *SLC2A3*, *BTG2*, *SOX4*, *OSM*, *SGK1*, *TGFBR3*, *OTUD1*, *COLQ* and *CCL5*). For the COMBAT and INCOV datasets, a similar approach was applied to calculate the IL2-AIS scores for each pseudo-bulk sample. Specifically, *z*-scores of normalised expression levels of the 41 signature genes were first calculated for each pseudo-bulk sample within each major cell type. The IL2-AIS scores were then derived as the sum of *z*-scores of upregulated signature genes, subtracted by that of downregulated signature genes. As the *z*-scores were calculated from samples within a dataset, one important limitation of this definition was that comparisons of IL2-AIS scores were only allowed within the same dataset, but not across different datasets.

### Modelling the dynamics of the IL2-AIS scores in the INCOV cohort

For the INCOV cohort, where multiple longitudinal samples are available for most individuals, we modelled the IL2-AIS scores using a Bayesian linear model to account for the inter-individual variation:

$${S}_{i,t} \sim \mathrm{Normal}\left({\mu }_{i,t}, \sigma \right)$$

$${\mu }_{i,t}= {\alpha }_{i}+ {\beta }_{t}$$

$${\alpha }_{i} \sim \mathrm{Normal}\left(0, 15\right)\;\mathrm{for}\;i=1..168$$

$${\beta }_{t} \sim \mathrm{Normal}\left(0, 10\right)\;\mathrm{for}\;t=1..7$$

$$\sigma \sim \mathrm{LogNormal}\left(0, 5\right)$$

where $S$ is the observed IL2-AIS scores, $i$ is the index of the individual and $t$ is the index of sampling time represented as a categorical variable with seven levels: 0–7 days, 8–14 days, 15–28 days, 29–60 days, 61–90 days, 91–150 days and ≥ 151 days. ${\alpha }_{i}$ and ${\beta }_{t}$ represent the individual-specific effect and the effect of sampling time, respectively. As the IL2-AIS score was formulated as the sum of *z*-scores of the 41 IL2-AIS genes, $\mathrm{E}(S)=0$. Therefore, an intercept term for ${\mu }_{i,t}$ was not included. We interpreted ${\alpha }_{i}$ as the time-adjusted IL2-AIS score of individual $i$ and ${\beta }_{t}$ as the expected IL2-AIS score given sampling time $t$. Regularising priors were used for ${\alpha }_{i}$ and ${\beta }_{t}$. Posterior mean values and 95% confidence intervals of parameters were estimated using a Markov Chain Monte Carlo approach implemented in Turing.jl [14].

### NanoString transcriptomic data analysis

The processed NanoString bulk transcriptomic data for the Gedda et al. (2022) cohort [7] was accessed from Gene Expression Omnibus [15, 16]. Among the 162 convalescent COVID-19 participants, 23 were excluded due to the lack of a precise date of onset of COVID-19 symptoms. All 40 healthy control participants were included. Among the 785 genes profiled in the dataset using the NanoString nCounter Human Host Response panel, 16 IL2-AIS genes were present, including 12 upregulated genes (*MYC*, *CXCR1*, *OAS1*, *TNFSF10*, *FASLG*, *CCR10*, *STAT1*, *CX3CR1*, *CD40LG*, *IL32*, *EOMES* and *CCR1*) and four downregulated genes (*OSM*, *SLC2A3*, *CXCR4* and *CCL5*). The IL2-AIS* scores were defined for each sample as the sum of *z*-scores of normalised transcription levels of upregulated IL2-AIS genes, subtracted by that of downregulated IL2-AIS genes.

### STRING network analysis

From the 1419 constituent genes of COMBAT Component 187, the top 50 genes with the highest loading scores were selected for STRING protein interaction network analysis [17]. Given the very strong correlation between the relative contribution of the same core set of genes to both the IL2-AIS and Component 187, the selection of the top 50 genes of Component 187 not only facilitated the visualisation of the gene network, but also allowed to focus on the main biological pathways contributing specifically to the IL2-AIS identified in this study. A gene network and pathway analysis on the full 1419 constituent genes of Component 187 is provided in the COMBAT study [5]. Physical and functional interactions identified from text mining, experimental evidence, annotated databases and co-expression were used. The minimum required interaction score was set to 0.15. The resulting protein interaction network can be accessed using the following link: https://version-11-5.string-db.org/cgi/network?networkId=bU31lXEkAFvX.

## Results

### COVID-19 patients display a pro-inflammatory gene expression signature

**Fig. 1:**
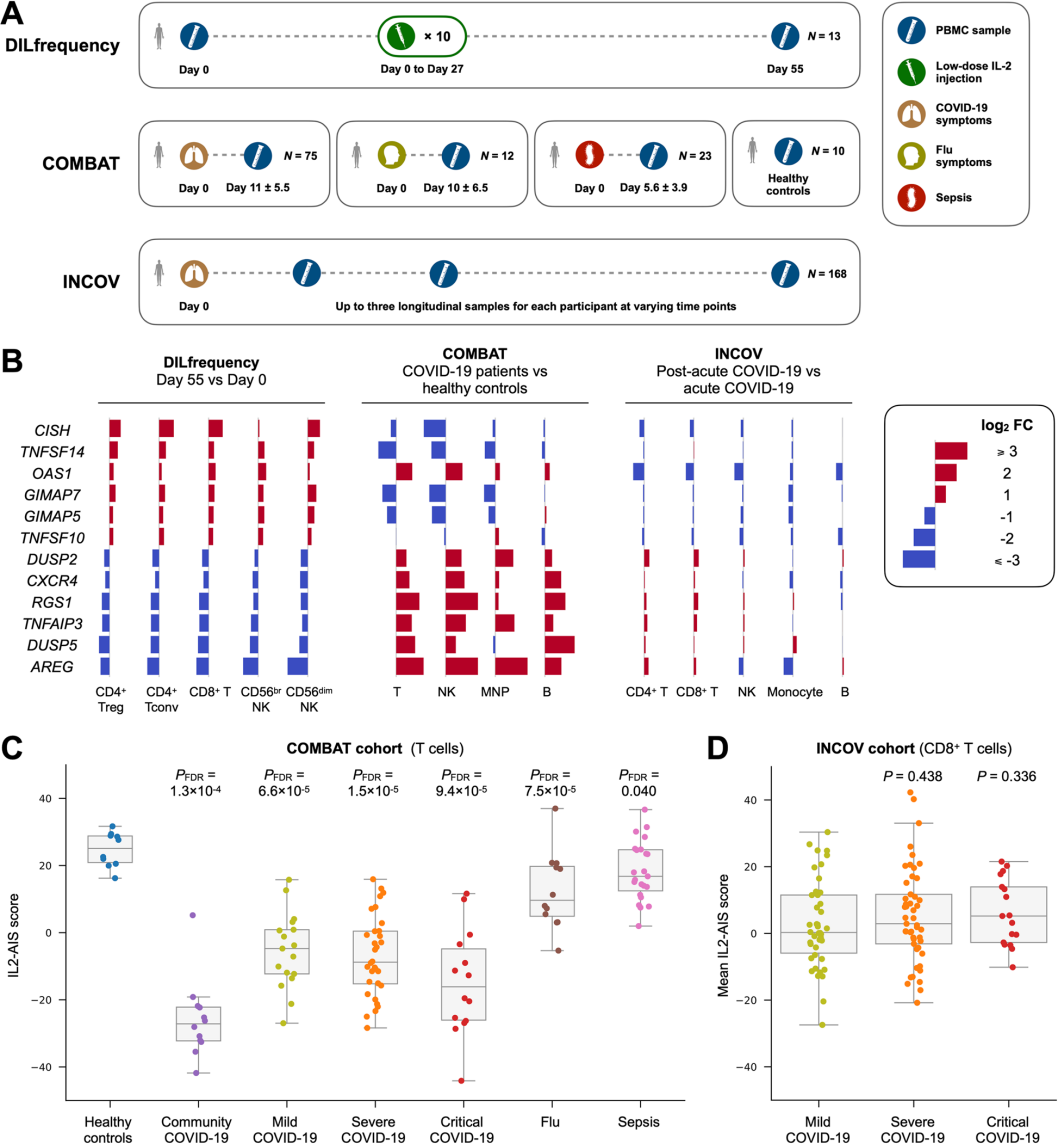
Low-dose IL-2 immunotherapy and SARS-CoV-2 infection induce opposite transcriptional changes in immune cells. **A** Overview of the DILfrequency, COMBAT and INCOV cohorts. **B** Differential expression induced by low-dose IL-2 immunotherapy (DILfrequency) and SARS-CoV-2 infection (COMBAT and INCOV) on the top six up and downregulated genes from the anti-inflammatory gene expression signature induced by low-dose IL-2 treatment (IL2-AIS), previously identified in the DILfrequency cohort. Data shown depicts the log_2_ fold change (FC) values in each cohort and were calculated in the different immune subsets identified in the respective study. *P* values and additional IL2-AIS constituent genes are shown in Additional file 3: Fig. S1. **C **Distribution of the IL2-AIS scores in T cells from each participant group in the COMBAT cohort. Data was stratified by disease group and COVID-19 disease severity. *P* values were calculated by comparing each patient group with healthy controls using a two-sided Mann–Whitney *U* test followed by FDR adjustment. **D** Mean IL2-AIS scores in CD8^+^ T cells from each participant group in the INCOV cohort from samples collected during the first 14 days after symptoms onset. Data was stratified by COVID-19 disease severity. *P* values are calculated by comparing the severe or critical COVID-19 group with the mild COVID-19 group using a two-sided Mann–Whitney *U* test. In **C** and **D**, each box ranges from the first quartile (Q1) to the third quartile (Q3), with a central line indicating the median. Treg, regulatory T cells. Tconv, conventional T cells. MNP, mononuclear phagocytes. The IL2-AIS scores were derived from pseudo-bulk samples aggregated from single-cell RNAseq data of PBMCs

Recently, we have characterised a prolonged transcriptional signature in peripheral blood from type 1 diabetic (T1D) patients treated with low-dose IL-2 (DILfrequency cohort; Fig. 1A) [4]. The transcriptional alterations were detected in all T and NK cell subsets analysed and displayed distinct anti-inflammatory characteristics, including upregulation of the negative regulator of cytokine signalling *CISH*. However, it is difficult to accurately predict the inflammatory context underlying the co-regulation of a large set of genes simply from assessing the directionality of expression, as many genes can have complex functions in vivo. Therefore, to extend the support for the physiological relevance and anti-inflammatory nature of the identified IL2-AIS, we examined the signature in a well-established inflammatory context in COVID-19 patients, using the COMBAT and INCOV datasets (Fig. 1A).

To investigate the specific regulation of the 41 identified IL2-AIS constituent genes in COVID-19 patients, we initially compared COVID-19 patients with healthy controls in the COMBAT cohort. We found that most IL2-AIS genes were regulated in the opposite direction in COVID-19 patients (Fig. 1B). For example, in contrast to the transcriptional changes induced by low-dose IL-2 in T1D patients, we observed a downregulation of *CISH* and an upregulation of *AREG* in COVID-19 patients during acute infection, across multiple immune cell types. This inverted expression of the IL2-AIS was specifically observed in COVID-19 patients, but not in the context of other severe pro-inflammatory conditions such as in hospitalised influenza or sepsis patients (Fig. 1C). Furthermore, we observed that the COVID-19-specific IL2-AIS gene expression profile was observed across all disease severity groups and was particularly pronounced in a sub-group of community COVID-19 cases (Fig. 1C). The community COVID-19 group consisted of a separate cohort of otherwise healthy healthcare workers infected by SARS-CoV-2, who had mild disease or no symptoms and were not admitted to hospital. Compared to the hospitalised COVID-19 patient groups, community COVID-19 patients were sampled later (7 or more days after the initial symptoms; average 13.8 days) upon returning to work. Of note, cell composition analysis revealed that the community COVID-19 patients were broadly comparable to healthy donors [5], indicating that they represent a very different patient group, sampled during the recovery phase. The lower IL2-AIS score in this group of recovered patients, but not between disease severity groups in the hospitalised patients (Fig. 1C), indicates that the induction of the IL2-AIS is not directly associated with the severity of the COVID-19 symptoms during the acute phase of the disease and is therefore not merely the reflection of an increased pro-inflammatory state during the acute phase of the disease. Our findings suggest that the lower expression of the IL2-AIS in community COVID-19 patients likely reflects a more general alteration of the transcriptional profile of cells after the onset of acute symptoms.

In support of a progressive induction of these gene expression changes following infection, we obtained consistent evidence for the induction of the IL2-AIS genes by comparing longitudinal samples in the INCOV dataset that were taken in the post-acute phase of COVID-19 with those taken in the acute phase (Fig. 1B). Although only some of the individual changes of each IL2-AIS gene reached statistical significance, they were consistently observed across all analysed immune cell types including T, B and NK cells and in monocytes (Additional file 3: Fig. S1).

### Pro-inflammatory gene expression profile is sustained in COVID-19 patients

**Fig. 2:**
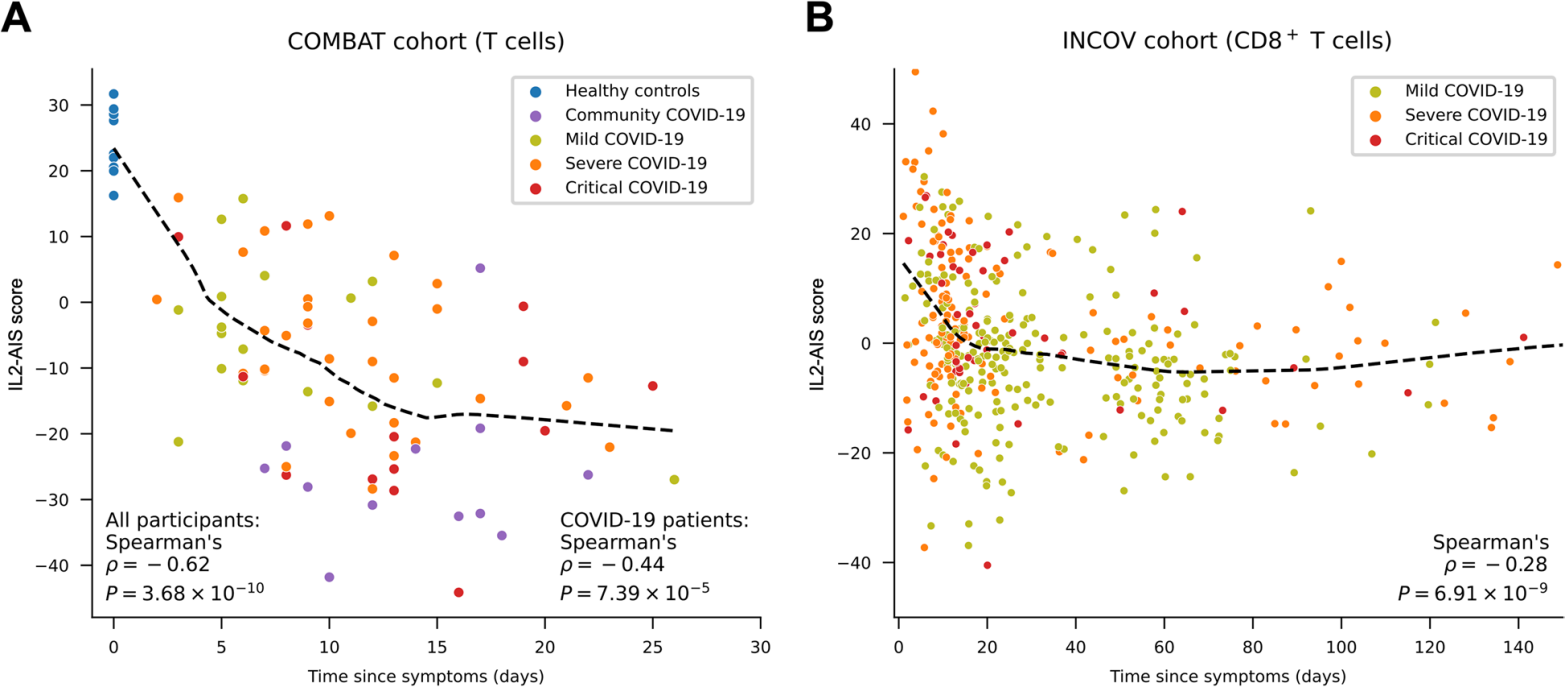
The IL-2-induced anti-inflammatory gene expression signature (IL2-AIS) score is progressively decreased after SARS-CoV-2 infection. **A**, **B** Decrease of IL2-AIS scores after the onset of symptoms in COVID-19 patients from the COMBAT cohort (**A**) or INCOV (**B**) cohorts. Each dot represents a clinical sample, and colours depict the different COVID-19 disease severity groups. In the INCOV cohort, patients are grouped by their worst recorded COVID-19 severity. The IL2-AIS score was calculated in T cells and CD8^+^ T cells in the COMBAT and INCOV cohorts, respectively. The variation in the IL2-AIS score from additional cell types are shown in Additional file 3: Fig. S2. IL2-AIS, anti-inflammatory gene expression signature induced by low-dose IL-2 immunotherapy. Dashed black lines represent locally weighted scatterplot smoothing (LOWESS) curves

Analysis of the dynamics of the transcriptional changes following SARS-CoV-2 infection in both COVID-19 cohorts revealed a progressive modulation of the IL2-AIS genes. These data are consistent with the establishment of a pro-inflammatory environment in COVID-19 patients, reflected in the reduction of the IL2-AIS score (Fig. 2 and Additional file 3: Fig S2). The induction of these transcriptional alterations was particularly pronounced for the first 1–2 weeks after the onset of symptoms and is consistent with the lower IL2-AIS score observed among community COVID-19 patients sampled later, during the recovery phase. However, we remark that even when accounting for the later sampling time, community COVID-19 patients still displayed a slightly lower IL2-AIS score (Fig. 2A), which may indicate unknown cohort-specific confounding factors contributing to these differences. An alternative explanation is that the increased induction of this set of genes in community COVID-19 patients reflects a resolved immune response to the viral infection in this group of otherwise healthy patients with mild or asymptomatic disease. The sustained increased expression of these genes could therefore reflect a period of heightened inflammatory responsiveness to further viral infection, which would be more pronounced in healthy individuals after viral clearance and very distinct from the transcriptional signatures associated with the exacerbated anti-viral inflammatory responses observed in hospitalised patients during the acute phase of the disease.

Based on available cell-type annotation data, we also investigated the dynamics of the IL2-AIS in specific cell subsets, which confirmed that almost all evaluated cell subsets displayed IL2-AIS score changes (Additional file 3: Fig. S3) similar to that observed for the aggregated cell types (Fig. 2 and Additional file 3: Fig. S2). This suggested that the IL2-AIS changes reflected differential expression within each cell subset, rather than relative compositional changes among different subsets.

In the INCOV cohort, follow-up samples taken several months after the initial symptoms showed that the alteration of the immune transcriptional landscape was maintained for approximately 3 to 4 months after the onset of symptoms (Fig. 2B). The dynamics of the transcriptional changes were largely consistent in all immune populations assessed in both COVID-19 cohorts, while the magnitude was largest in T and NK cells and smallest in B cells (Additional file 3: Fig. S2). Considering that most participants in the INCOV cohort were sampled multiple times after symptom onset, we modelled the participant-specific effects and time-specific effects to the IL2-AIS scores in the INCOV cohort (see Methods), which confirmed the progressive decline during the first 90 days post-infection in CD4^+^ T, CD8^+^ T and NK cells, followed by a slow recovery (Additional file 3: Fig. S4). In monocytes and B cells, this pattern was less clear, though a general trend of decline was evident (Additional file 3: Fig. S4).

The longevity of the transcriptional changes and the specificity towards COVID-19 infection highlights the potential impairment of the immune response in post-acute COVID-19 patients, which could underpin the manifestation of PASC. In agreement with this hypothesis, we found that severe and critical COVID-19 patients in the INCOV cohort who reported one or more PASC symptoms had lower time-adjusted IL2-AIS scores compared to those reporting no PASC symptoms (Additional file 3: Fig. S3), which is consistent with more pronounced inflammatory responses in patients suffering from PASC. However, although this difference was consistently observed in all analysed immune subsets, the effect size was small and statistical significance was not reached in all cell types (Additional file 3: Fig. S3), likely due to the heterogeneity of PASC symptoms [6]. Further dissecting the PASC symptoms into specific categories, we found that the IL2-AIS scores appeared to be most strongly correlated with viral respiratory and neurological symptoms, though none of the specific analyses reached statistical significance after correcting for multiple comparisons (Additional file 3: Fig. S5). In all three datasets analysed, we found that the IL2-AIS score was not correlated with age or sex (Additional file 3: Fig. S6), although both factors were found to be associated with COVID-19 severity and PASC symptoms [18, 19].

To obtain further evidence of these transcriptional changes, we assessed data from a recent study published by Gedda et al. [7], using the NanoString nCounter platform to profile the transcriptional landscape of red blood cell-depleted whole blood samples taken from 162 convalescent COVID-19 patients and 40 healthy controls (Additional file 3: Fig. S7A). The NanoString nCounter Human Host Response panel used in this study included 16 of the 41 IL2-AIS genes, allowing us to derive a signature score (IL2-AIS* score) representing a subset of the original IL2-AIS. Consistent with our previous observations, the IL2-AIS* score showed a progressive decrease during the first 2 months after infection, followed by a gradual recovery towards the baseline level measured in healthy control participants (Additional file 3: Fig. S7B). Despite the limited overlap between the IL2-AIS genes and the NanoString transcriptional panel, as well as differences in cell types, the consistency observed in the Gedda et al. cohort provides additional validation of the longevity of the transcriptional alterations induced by SARS-CoV-2 infection.

### Transcriptional alterations in COVID-19 patients are associated with the transcription factor NF-κB

**Fig. 3:**
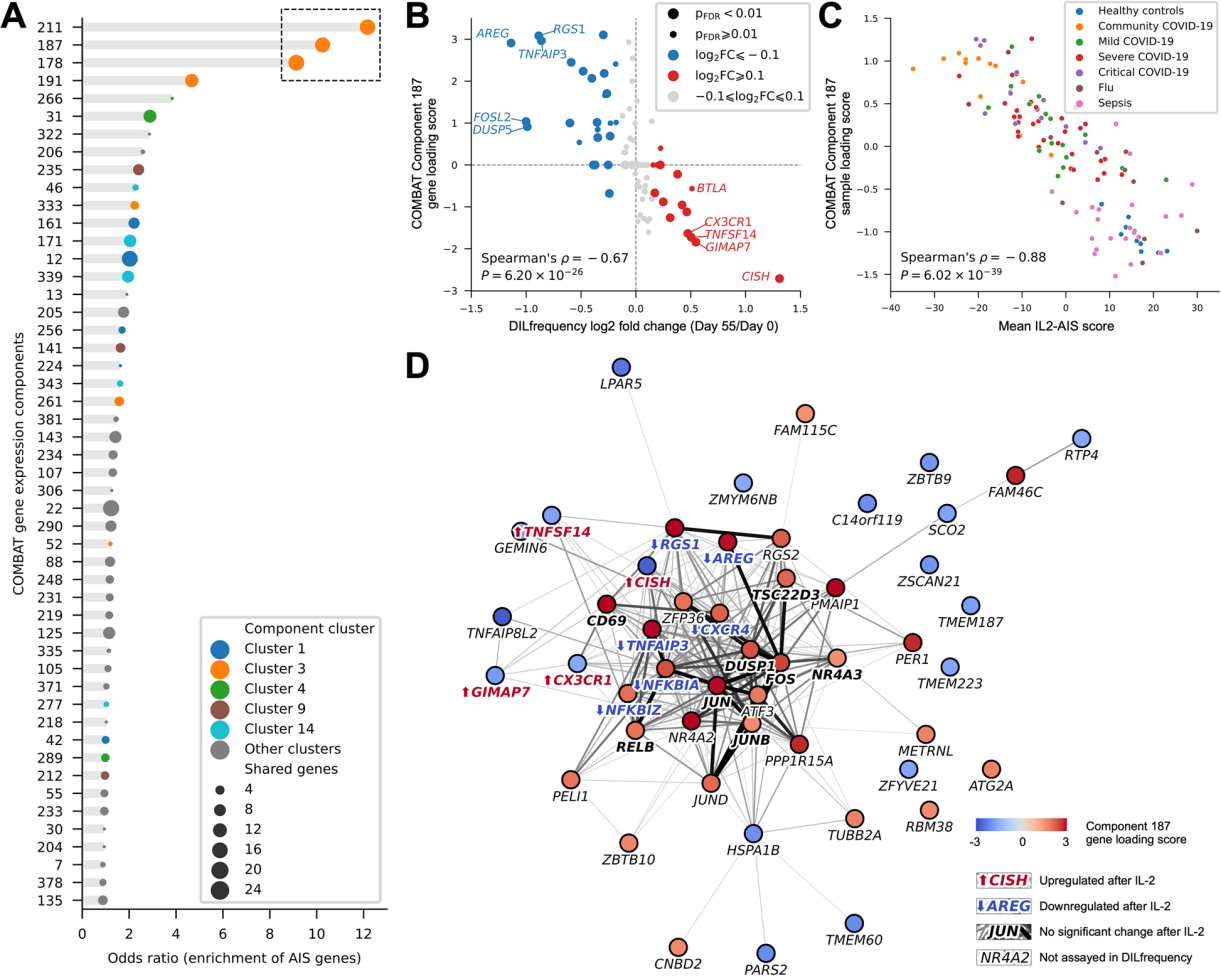
NF-kB is associated with the regulation of the transcriptional alterations of IL2-AIS genes in COVID-19 patients. **A** Enrichment of IL2-AIS constituent genes in each multi-modal gene expression component reported in the COMBAT study [5]. Each dot represents a disease-associated gene expression component identified in the COMBAT study. Dot sizes represent the number of shared genes between the respective component and the 41 IL2-AIS constituent genes. The top 50 components are shown, ranked by their odds ratios of enrichment (*x*-axis). Colours depict cluster membership as reported in the COMBAT study. Components in the same cluster are associated with diseases in a similar way. Cluster 3 is associated with all severity groups of COVID-19. The three components with odds ratio ⩾ 5 are highlighted with a dashed blacked box. **B** Component 187 loading scores are negatively correlated with the differential expression induced by IL-2 immunotherapy, as reported in the DILfrequency cohort. Of the 1,419 genes included in Component 187, 69 are also present in the target transcriptional panel used in the DILfrequency study [4]. The effects of IL-2 immunotherapy and SARS-CoV-2 infection on the induction of these genes are compared by correlating component gene loading score (*y*-axis) with log_2_ fold change (*x*-axis) in the CD8 population. The positive and negative values in Component 187 loading scores represent the upregulation and downregulation of the corresponding gene, respectively, after SARS-CoV-2 infection. Each dot represents a gene. Larger dots represent genes with FDR-adjusted *P* values < 0.01 in the DILfrequency dataset. **C** Correlation between the IL2-AIS score and the Component 187 sample loading score. Each dot represents a participant in the COMBAT cohort and colours depict the different participant groups. **D** STRING [17] protein interaction network of top 50 genes in Component 187. Each node represents a gene. Node colours represent Component 187 gene loading scores. IL2-AIS genes are labelled in bold text with directions of IL-2-induced differential expression shown in arrows. Each edge represents experimental or inferred protein–protein interaction between two genes. Edge widths and colours represent interaction scores, with thicker lines and darker colours representing higher scores. IL2-AIS, anti-inflammatory gene expression signature induced by low-dose IL-2 immunotherapy

As the IL2-AIS was initially identified in the DILfrequency study using a targeted panel of 565 transcripts designed for profiling T and NK cells [4], we sought to understand the observed changes in the broader transcriptional landscape of immune cells in COVID-19 patients. In the COMBAT study, the authors employed a multi-parametric tensor decomposition analysis combining gene expression, surface protein expression (CITE-seq), plasma proteomics and cell subset abundance (flow cytometry) data, to identify 130 COVID-19-associated gene expression components categorised into 14 clusters, with each component containing a large number of weighted genes likely differentially expressed in COVID-19 patients [5]. We analysed whether IL2-AIS genes, compared to other genes assayed in the DILfrequency study, were enriched in specific components. We found three similar components in Cluster 3 (Component 211, Component 187 and Component 178), each showing strong enrichment of the 41 IL2-AIS genes (odds ratio > 8; Fig. 3A). From these three components in Cluster 3, Component 187 was highlighted by the authors as the most significant COVID-19-specific signature out of all the identified signatures (FDR-adjusted *P* = 6.74 × 10^−15^) [5]. Furthermore, we found that among the signatures associated with COVID-19 disease severity, Component 187 was unique in the longevity of the signature (Additional file 3: Fig. S8). In contrast to Component 187, the other COVID-19-associated Components were more strongly modulated immediately after the onset of symptoms, indicating virus-induced alterations in cell composition and activation state during the acute phase of the disease — as illustrated for example by the classical monocyte-derived type 1 interferon (IFN) signature (Component 235; Additional file 3: Fig. S8).

Of the 1419 genes contributing to Component 187, 77 were present in the transcriptional panel used in the DILfrequency study. We found that virtually all genes upregulated in IL2-AIS had negative loading scores in Component 187 and vice versa (Fig. 3B), indicating a strong inverse correlation between the differential gene expression induced by IL-2 treatment in T1D patients and the response to infection in COVID-19 patients. Correspondingly, we observed a strong negative correlation between the mean Component 187 sample loading score and the mean IL2-AIS score among COMBAT participants (Fig. 3C), indicating that Component 187 and the IL2-AIS likely shared common underlying mechanisms.

Given the strong correlation between these two transcriptional signatures, we next performed a network analysis on the top 50 genes of Component 187 to gain some insight into the putative biological mechanism underpinning the identified IL2-AIS. This analysis suggested a central role of the transcription factor NF-κB on the regulation of this transcriptional programme, as evidenced by a number of target genes previously shown to be modulated by NF-κB in SARS-CoV-2-infected CD8^+^ T cells, including *NFKBIA*, *NFKBIZ*, *TNFAIP3* and *CXCR4* (Fig. 3D) [20]. We observed a markedly reversed regulation of the core genes of this NF-κB-regulated transcriptional network in the context of COVID-19 versus low-dose IL-2 treatment in T1D (Fig. 3D). Furthermore, we also observed a significant enrichment of known NF-kB target genes (https://www.bu.edu/nf-kb/gene-resources/target-genes/) within the top 50 genes of Component 187, including *RELB*, *NR4A2*, *DUSP1*,* CD69* and the AP-1 transcription factor complex genes (*FOS*, *FOSB*, *JUN*, *JUNB* and *JUND*). However, we note that these core NF-kB target genes, including the AP-1 genes, were not identified in the IL2-AIS (Fig. 3D), suggesting that they were not significantly modulated by low-dose IL-2 therapy and are specifically modulated by SARS-CoV-2 infection. Notably, all identified NK-kB target genes show positive Component 187 loading scores (Fig. 3D), suggesting that increased expression of early response factors such as NF-kB and the transcription factor complex AP-1 is involved in sustaining the pro-inflammatory gene expression profile in circulating immune cells during the post-acute phase of COVID-19.

## Discussion

The unprecedented breath of high-dimensional datasets generated in cohorts of COVID-19 patients has provided a unique resource to investigate the transcriptional profile of immune cell populations in response to infection. In the present study, we show that SARS-CoV-2 infection leads to a long-lived alteration of the transcriptional landscape of immune cells in blood for over 2 months after the onset of the clinical symptoms. We show that a core set of co-regulated immune response genes display a pronounced and specific inverted expression profile from original discovery in an anti-inflammatory context (T1D patients treated with low-dose IL-2) compared to COVID-19 patients.

A key finding was the observation that the transcriptional changes in COVID-19 patients were associated with the activation of the NF-kB signalling pathway, which is consistent with its previously identified role in severe COVID-19 patients [21]. This was supported by the upregulation of the NF-kB inhibitor genes *NFKBIA* and *NFKBIZ*, the AP-1 transcription factor complex genes *FOS*, *JUN*, *FOSB* and *JUNB* as well as other NF-kB target genes such as *TNFAIP3*, *RELB*, *NR4A2*, *DUSP1* and *CD69* in response to infection. In agreement with this hypothesis, a recent study identified a specific transcriptional profile in COVID-19 patients that was consistent with NF-kB-driven inflammation in these patients, which was not observed in patients infected with influenza [22]. In contrast, the influenza patients displayed a much stronger type I interferon (IFN) response, leading to the upregulation of the canonical IFN transcriptional signature [22]. Furthermore, in a ciliated cell line model, a very similar NF-kB-driven transcriptional signature (including *NFKBIA*, *FOS* and *JUN*) has been shown to be specifically induced in SARS-CoV-2-infected cells immediately (< 3 h) post-infection [23]. Notably, this gene signature was not observed in either uninfected or bystander cells, indicating a very specific upregulation of the NF-kB signalling pathway in SARS-CoV-2 infected cells. Further supporting this hypothesis, the upregulation of an NF-kB signature, including *TNFAIP3*, *NFKBIA* and *FOS*, was also induced in an epithelial cell line model 8 h after infection with SARS-CoV-2 [24].

Currently, the exact mechanism underlying the observed transcriptional changes is unknown. It is well established that during the acute phase of the disease, some COVID-19 patients display higher levels of several classical pro-inflammatory cytokines, including IL-6, IFNa and TNF [5, 25]. It is plausible that the establishment of a pro-inflammatory environment associated with the disease symptoms can have a more prolonged effect on the immune transcriptional profile. However, the signalling pathways associated with these classical pro-inflammatory cytokines are complex, and there are often synergistic effects between them; particularly in a scenario such as COVID-19, where many of these signalling factors are present. Nevertheless, we provide evidence for a prolonged alteration of a systematic set of co-regulated genes that can be detected months after the resolution of acute symptoms. The long-term alteration of the immune system during the post-acute phase of the disease is usually asymptomatic, but it could affect the likelihood of PASC development, a possibility that needs further study. Recently, there has been considerable interest in understanding better this critical phase of the disease given the rising number of patients presenting with PASCs, and similar long-term effects of infection have been recently reported in the context of the response to further pro-inflammatory insults, such as influenza infection [26]. Our study supports the hypothesis that the transcription factor NF-kB plays a critical role in the establishment and maintenance of the observed transcriptional alteration. Notably, it is known that NF-kB signalling is involved in several of the classical pro-inflammatory signalling cytokine pathways. Further work is therefore warranted to better understand the exact mechanism and combination of factors that contribute to the prolonged activation of the NF-kB signalling pathway observed in this study.

The longevity of the transcriptional alterations induced by SARS-CoV-2 infection suggests that the inflammatory environment is more prolonged than the period of increased levels of pro-inflammatory cytokines in plasma, associated with the symptomatic phase of the disease. One hypothesis is that the cytokines produced during the acute phase of the disease have a longer period of biological activity in tissues. This could be caused by the maintenance of higher local concentrations of cytokines in the site of infection and associated immune tissues through the binding of cytokines to the extracellular matrix. Several pro-inflammatory cytokines, most notably TNF, have been previously shown to bind to the extracellular matrix [27–29], which could promote a much longer period of biological activity in tissues, thereby inducing the transcriptional signature detected in this study. A long-lasting immune alteration that also possibly reflects a continuing inflammatory stimulus or long-lived pro-inflammatory cytokines on the extracellular matrix has been reported in mild COVID-19 patients, with infection inducing a pro-inflammatory response in monocyte-derived macrophages that continues to be detected 3–5 months following SARS-CoV-2 infection [30]. In a separate study, pro-inflammatory markers such as IL-8 and sTIM-3 were still elevated 4 months after the cessation of COVID-19 symptoms in patients having had mild or moderate disease, but the increases resolved 8 months post-infection, except in patients with long COVID [31]. These results support the establishment of a prolonged pro-inflammatory environment following SARS-CoV-2 infection. Given the extended period in which these alterations can be detected, it will be particularly important to measure the cumulative effect of multiple infections on the induction of this signature to investigate whether this could represent a pathogenic mechanism underlying the increased risk of mortality and burden of post-acute sequelae recently observed in patients re-infected with SARS-CoV-2 [2].

A corollary of our results is that low-dose IL-2 immunotherapy could have a therapeutic application to revert this prolonged period of immune dysfunction in recovered COVID-19 patients and reduce the occurrence of PASC. IL-2 has been shown to bind to the extracellular matrix [27], potentially promoting prolonged increased Treg fitness and causing the systemic decreased production/accumulation of pro-inflammatory cytokines in tissues, attributes that could have clinical applications. This putative preventative application of low-dose IL-2 is further supported by the strong safety record in patients — including children — undergoing long-term treatment [32]. Moreover, low-dose IL-2 treatment in SLE patients has been previously shown to lead to a threefold reduction in the incidence of both viral and bacterial infection [33], potentially reflecting the prolonged heightened anti-inflammatory state in these patients. However, a limitation of our study is that the observed transcriptional signatures were identified in patients with two very distinct pathophysiological manifestations (T1D and acute phase COVID-19). Therefore, we cannot be certain that the transcriptional alterations induced by low-dose IL-2 would be consistent in both settings. To mitigate the potential variability introduced by chronic immune activation in COVID-19 patients, it is critical to consider that the putative administration of low-dose IL-2 in COVID-19 patients should be restricted to the convalescent phase of the disease, long after the acute inflammation has been resolved, to avoid unwanted hyperactivation of the immune response during the acute phase of the disease. To date, there have been very few pilot studies investigating the use of low-dose IL-2 in COVID-19 patients, and these have been limited to the treatment of patients with acute disease, which may explain the reported lack of clear therapeutic benefit for these patients [34, 35]. Further work will therefore be necessary to support the potential clinical application of low-dose IL-2 in convalescent COVID-19 patients, including the retrospective analysis of the incidence of PASC in cohorts of patients treated with low-dose IL-2.

## Conclusions

Taken together, our findings shed new light into the convalescence period in COVID-19 patients and reveal long-lasting alterations to the transcriptional landscape of circulating immune cells that are consistent with a heightened pro-inflammatory state. The reversed pattern of gene expression compared to that induced by treatment with low-dose IL-2 immunotherapy in T1D [4] suggests that interval dosing of low-dose IL-2 for a month promotes a prolonged regulatory environment, which could potentially hasten the restoration of normal immune homeostasis in recovered COVID-19 patients. These results indicate the need to further investigate the mechanistic alterations induced by SARS-CoV-2 infection to better understand how to reduce the occurrence of long COVID-19 complications.

### Supplementary Information

**Additional file 1: Table S1.** Baseline characteristics of study participants. Participants excluded from this study are not shown. For the INCOV cohort, participants are grouped by their maximum COVID-19 severity across all measured time points. NA, not available.

**Additional file 2: Table S2.** Summary statistics of total mRNA counts of pseudo-bulk samples. Median and interquartile range (IQR) of total mRNA counts in each group of pseudo-bulk samples in the COMBAT and INCOV cohorts, and the number of excluded samples in each group.

**Additional file 3: Fig. S1.** COVID-19 patients from the COMBAT and INCOV cohorts display consistent differential expression of the IL2-AIS constituent genes but in the opposite direction. **Fig. S2.** Consistent decrease of IL2-AIS score after COVID-19 infection in multiple cell types. **Fig. S3.** IL2-AIS changes are consistent across various specific cell subsets. **Fig. S4.** Modelling the dynamics of the IL2-AIS scores. **Fig. S5.** IL2-AIS scores are correlated with post-acute sequelae symptoms in severe and critical COVID-19 patients. **Fig. S6.** The IL2-AIS score is not associated with age or sex. **Fig. S7.** Replicating a subset of IL2-AIS using the NanoString nCounter transcriptomics platform. **Fig. S8.** Temporal patterns of selected gene expression components in the COMBAT dataset.

## Data Availability

For the DILfrequency study, the single-cell sequencing data are available from NCBI Gene Expression Omnibus (GEO) under the accession number GSE201197 (https://www.ncbi.nlm.nih.gov/geo/query/acc.cgi?acc=GSE211378) [8]. Basic information about the study participants is shown in Additional file 1: Table S1. For the COMBAT cohort, the single-cell sequencing data and basic information about study participants are available from Zenodo (https://doi.org/10.5281/zenodo.6120249) [9]. For the INCOV cohort, the single-cell sequencing data are available from ArrayExpress under the accession number E-MTAB-10129 (https://www.ebi.ac.uk/biostudies/arrayexpress/studies/E-MTAB-10129?query=E-MTAB-10129) [10]. Basic information about the study participants is available from the relevant publication [6]. For the Gedda et al. (2022) cohort, the processed NanoString nCounter transcriptomics data are available from NCBI Gene Expression Omnibus under the accession numbers GSE211378 (https://www.ncbi.nlm.nih.gov/geo/query/acc.cgi?acc=GSE211378) [15] and GSE211394 (https://www.ncbi.nlm.nih.gov/geo/query/acc.cgi?acc=GSE211394) [16].

## Abbreviations

COMBAT: Oxford COVID-19 Multi-omics Blood Atlas
COVID-19: Coronavirus disease 2019
FACS: Fluorescence-activated cell sorting
FDR: False discovery rate
ICU: Intensive care unit
IFN: Type I interferon
IL: Interleukin
IL2-AIS: Anti-inflammatory signature induced by low-dose IL-2 treatment
NK: Natural killer
PASC: Post-acute sequelae of COVID-19
PBMC: Peripheral blood mononuclear cell
PMA: Phorbol myristate acetate
RNA-seq: RNA-sequencing
SARS-CoV-2: Severe acute respiratory syndrome coronavirus 2
T1D: Type 1 diabetes
Tconv: CD4^+^ conventional T cell
TNF: Tumour necrosis factor
Treg: CD4^+^ regulatory T cell
