# Supplementary material for: SARS-CoV-2 infection induces a long-lived pro-inflammatory transcriptional profile
Source: Genome Med. 2023 Sep 12;15:69. doi: 10.1186/s13073-023-01227-x (PMC10498514; doi:10.1186/s13073-023-01227-x)
Supplement: Supplementary file 3 — Additional file 3: Fig. S1. COVID-19 patients from the COMBAT and INCOV cohorts display consistent differential expression of the IL2-AIS constituent genes but in the opposite direction. Fig. S2. Consistent decrease of IL2-AIS score after COVID-19 infection in multiple cell types. Fig. S3. IL2-AIS changes are consistent across various specific cell subsets. Fig. S4. Modelling the dynamics of the IL2-AIS scores. Fig. S5. IL2-AIS scores are correlated with post-acute sequelae symptoms in severe and critical COVID-19 patients. Fig. S6. The IL2-AIS score is not associated with age or sex. Fig. S7. Replicating a subset of IL2-AIS using the NanoString nCounter transcriptomics platform. Fig. S8. Temporal patterns of selected gene expression components in the COMBAT dataset. [file 13073_2023_1227_MOESM3_ESM.pdf]

Additional file 3: Supplementary Figures

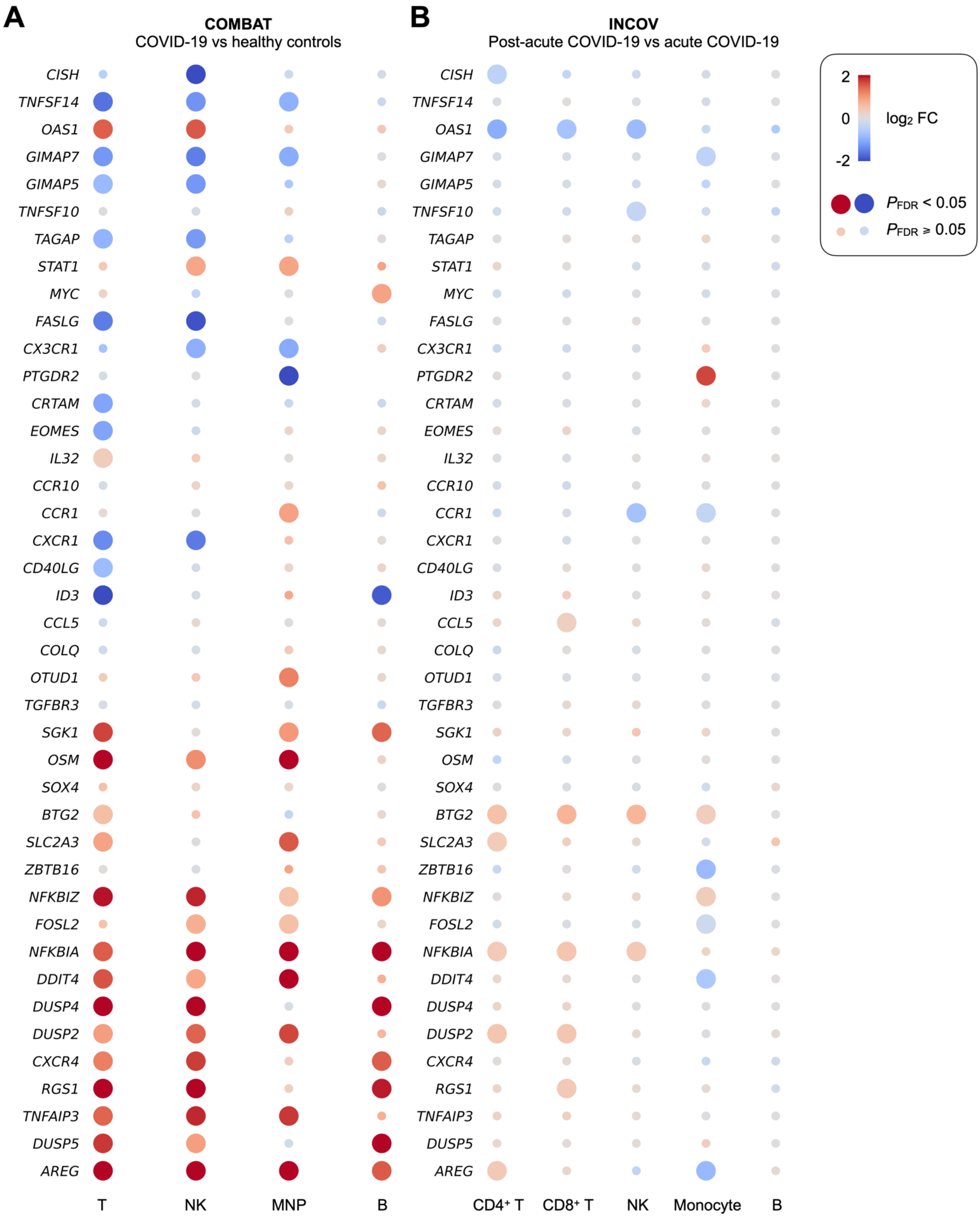

A
COMBAT
COVID-19 vs healthy controls
B
INCOV
Post-acute COVID-19 vs acute COVID-19
CISH
TNFSF14
OAS1
GIMAP7
GIMAP5
TNFSF10
TAGAP
STAT1
MYC
FASLG
CX3CR1
PTGDR2
CRTAM
EOMES
IL32
CCR10
CCR1
CXCR1
CD40LG
ID3
CCL5
COLQ
OTUD1
TGFBR3
SGK1
OSM
SOX4
BTG2
SLC2A3
ZBTB16
NFKBIZ
FOSL2
NFKBIA
DDIT4
DUSP4
DUSP2
CXCR4
RGS1
TNFAIP3
DUSP5
AREG
T
NK
MNP
B
CD4+ T
CD8+ T
NK
Monocyte
B
2
0
-2
log2 FC
PFDR < 0.05
PFDR ≥ 0.05

Figure S1. COVID-19 patients from the COMBAT and INCOV cohorts display consistent differential expression of the IL2-AIS constituent genes but in the opposite direction

A-**B**, Differential expression of all 41 IL2-AIS constituent genes previously reported in in the COMBAT (**A**) or INCOV cohorts (**B**). Differential expression (depicted as log2 fold changes) were calculated by comparing the expression between COVID-19 patients from all disease severity groups with healthy controls in COMBAT, and by comparing post-acute phase (29 to 84 days post symptoms) to acute phase (1-14 days post symptoms) COVID-19 participants in INCOV. The differential expression was performed on each immune cell subset identified in the respective study. Larger dots represent genes with FDR-adjusted $P < 0.05$. All 41 IL2-AIS genes exhibiting prolonged differential expression in the DILfrequency cohort are shown. See also Fig. 6a in ref. [4].

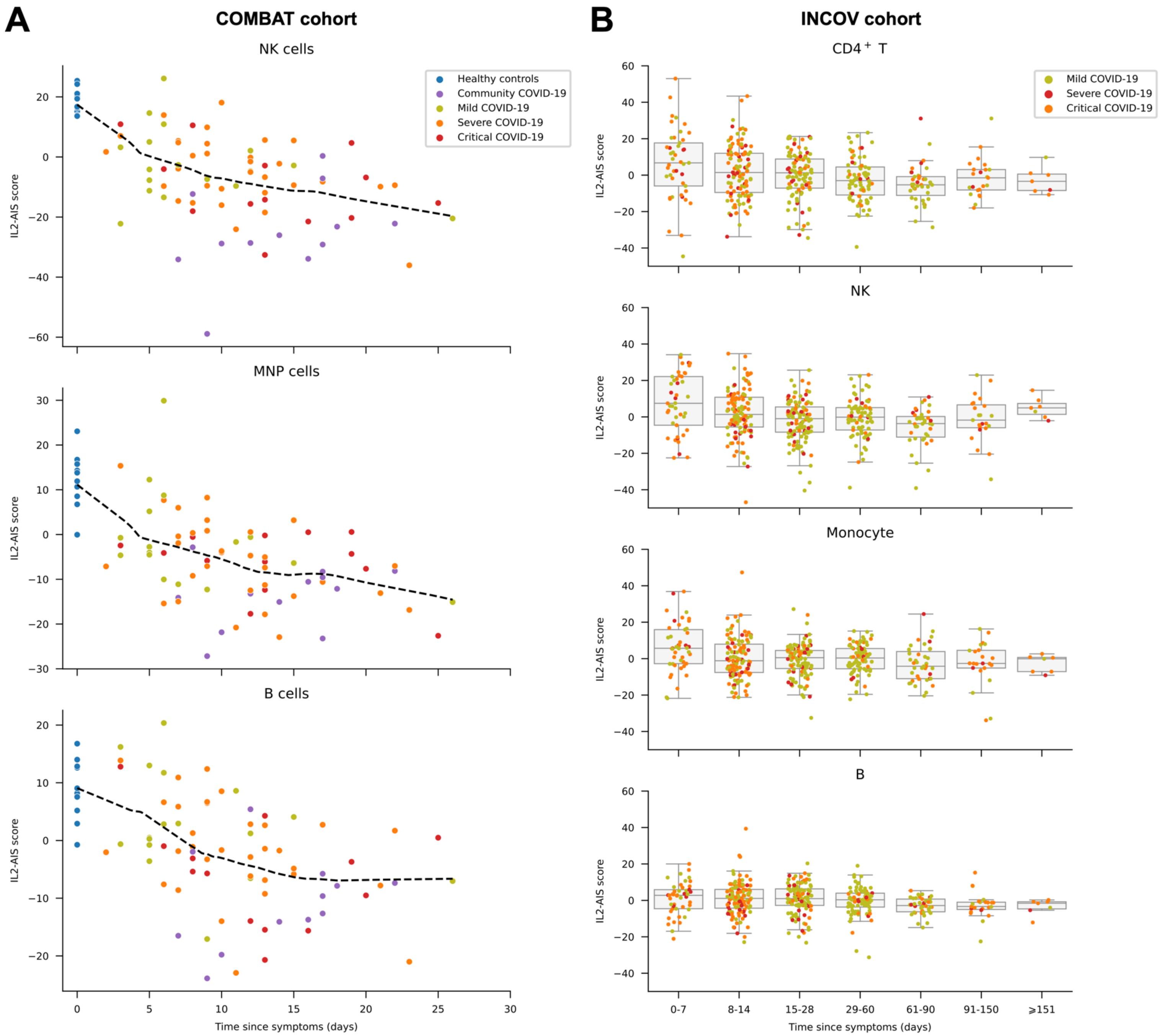

Figure S2. Consistent decrease of IL2-AIS score after COVID-19 infection in multiple cell types

A-B, Decrease of IL2-AIS scores after the onset of symptoms in COVID-19 patients from the COMBAT cohort (**A**) or INCOV (**B**) cohorts. Data shown represents the variation of the IL2-AIS scores from the identified NK, mononuclear phagocytes (MNP) and B cell subsets in COMBAT and from the $CD4^+$ T, NK, Monocyte and B cell subsets in INCOV. Each dot represents a clinical

sample, and colours depict the different COVID-19 disease severity groups. In the INCOV cohort, patients are grouped by their worst recorded COVID-19 severity. In **A**, the dashed black line represents locally weighted scatterplot smoothing (LOWESS) curves. In **B**, each box ranges from the first quartile (Q1) to the third quartile (Q3), with a central line indicating the median.

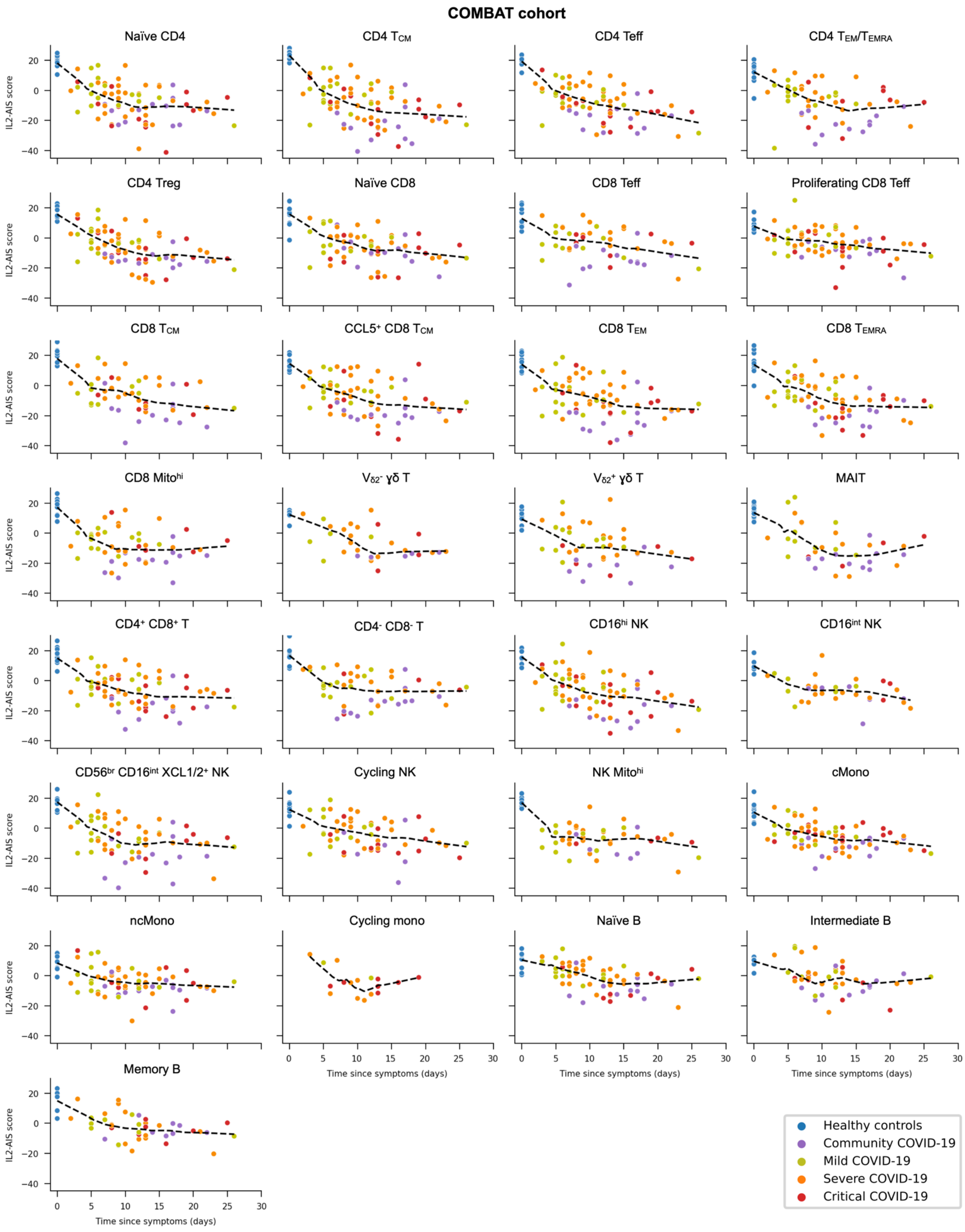

COMBAT cohort
Naïve CD4
CD4 TCM
CD4 Teff
CD4 TEM/TEMRA
CD4 Treg
Naïve CD8
CD8 Teff
Proliferating CD8 Teff
CD8 TCM
CCL5+ CD8 TCM
CD8 TEM
CD8 TEMRA
CD8 Mitohi
Vδ2- γδ T
Vδ2+ γδ T
MAIT
CD4+ CD8+ T
CD4- CD8- T
CD16hi NK
CD16int NK
CD56br CD16int XCL1/2+ NK
Cycling NK
NK Mitohi
cMono
ncMono
Cycling mono
Naïve B
Intermediate B
Memory B
IL2-AIS score
Time since symptoms (days)
Healthy controls
Community COVID-19
Mild COVID-19
Severe COVID-19
Critical COVID-19

Figure S3. IL2-AIS changes are consistent across various specific cell subsets.

Decrease of IL2-AIS scores after the onset of symptoms in COVID-19 patients from the COMBAT cohort, shown in 29 cell subsets for which at least 100 pseudo-bulk samples are available. Each dot represents a pseudo-bulk sample. Colours depict the different COVID-19 disease severity groups. Dashed black lines represent locally weighted scatterplot smoothing (LOWESS) curves.

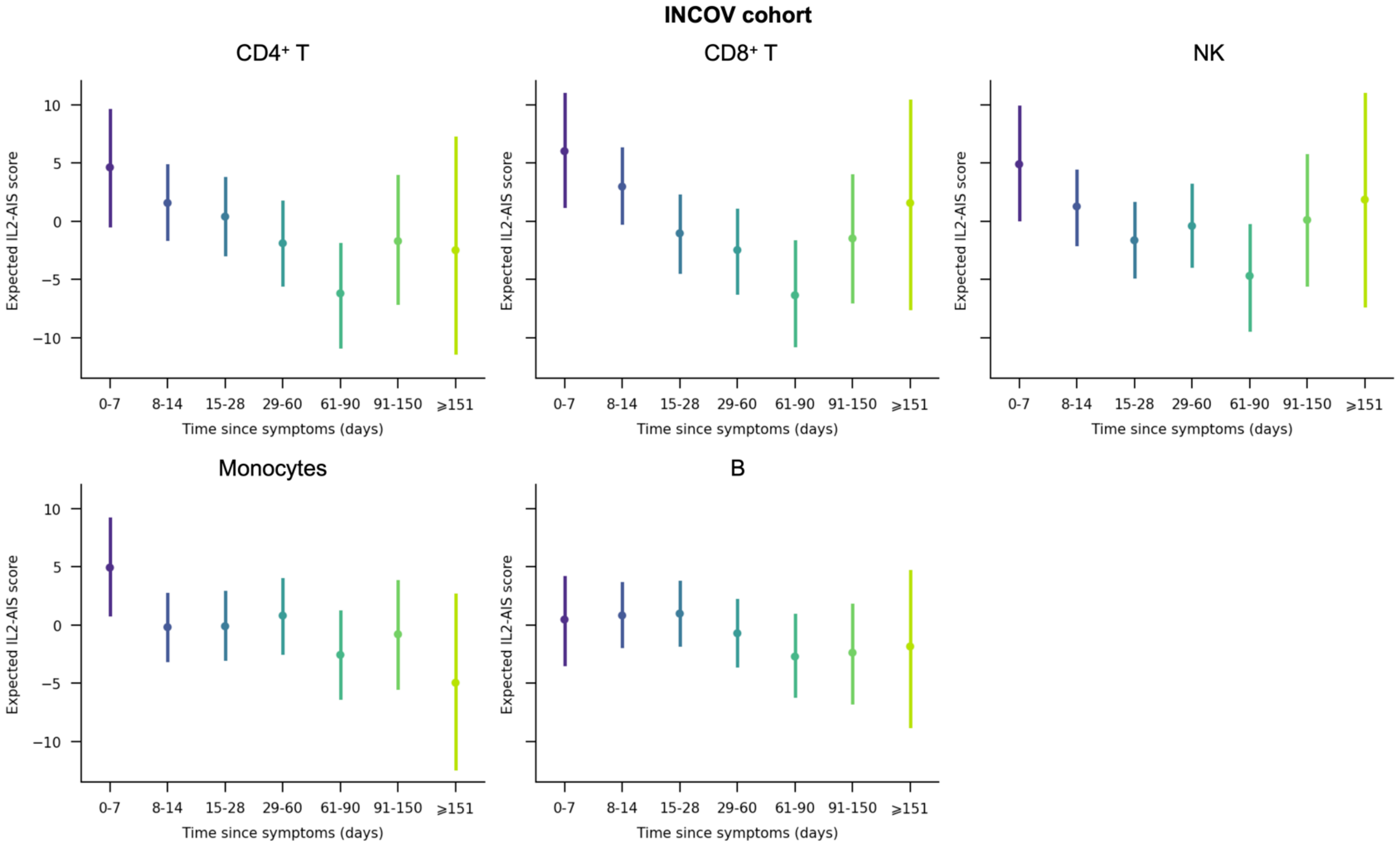

Figure S4. Modelling the dynamics of the IL2-AIS scores.

Posterior mean values (dots) and 95% confidence intervals (error bars) of the expected IL2-AIS scores (y axis) for each time range (x axis) estimated using a Bayesian linear model (**Methods**) for each cell type in the INCOV cohort.

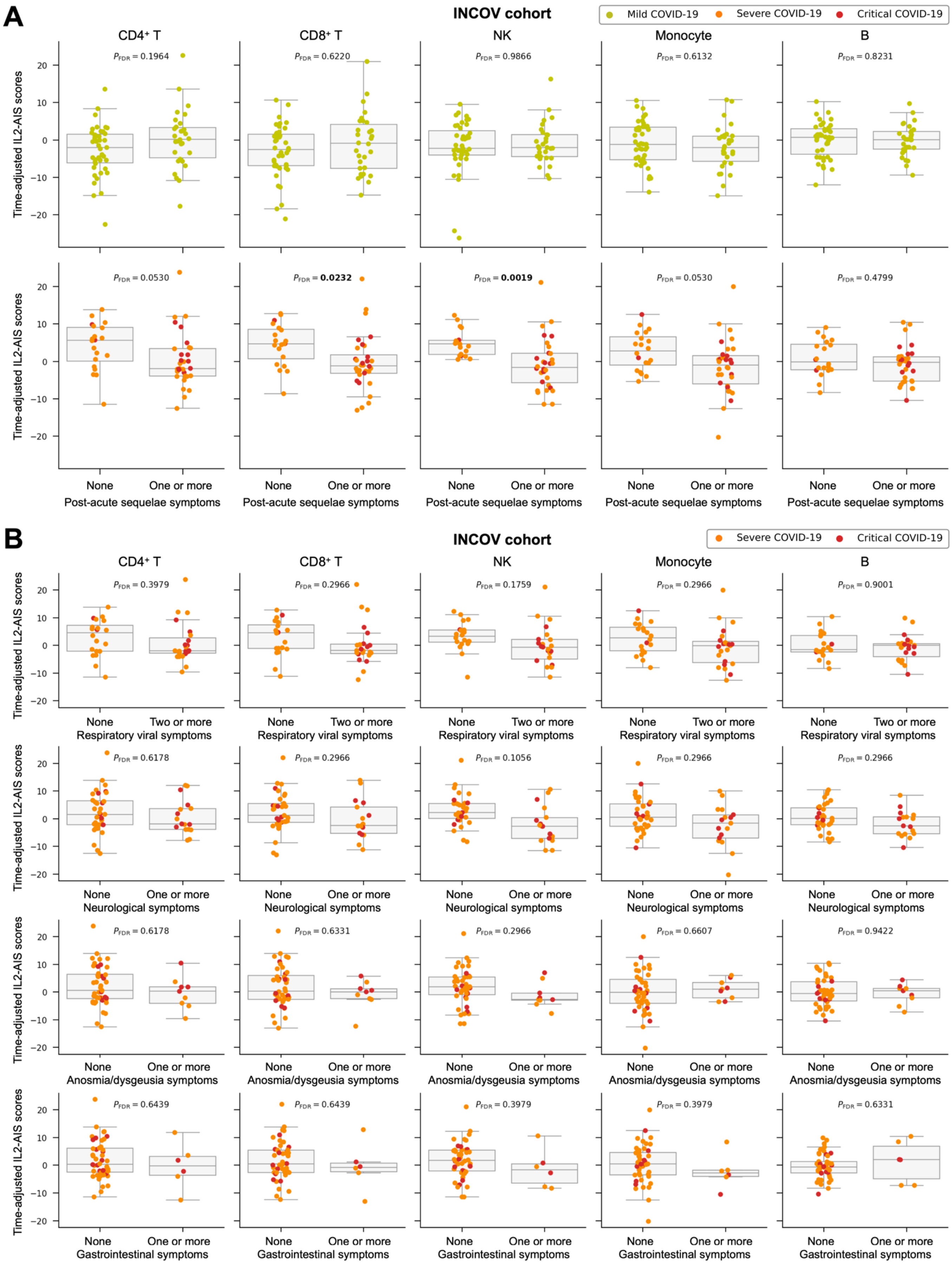

A
INCOV cohort
Mild COVID-19
Severe COVID-19
Critical COVID-19
CD4⁺ T
CD8⁺ T
NK
Monocyte
B
Time-adjusted IL2-AIS scores
P_FDR = 0.1964
P_FDR = 0.6220
P_FDR = 0.9866
P_FDR = 0.6132
P_FDR = 0.8231
P_FDR = 0.0530
P_FDR = 0.0232
P_FDR = 0.0019
P_FDR = 0.0530
P_FDR = 0.4799
None
One or more
Post-acute sequelae symptoms
B
INCOV cohort
Severe COVID-19
Critical COVID-19
CD4⁺ T
CD8⁺ T
NK
Monocyte
B
P_FDR = 0.3979
P_FDR = 0.2966
P_FDR = 0.1759
P_FDR = 0.2966
P_FDR = 0.9001
None
Two or more
Respiratory viral symptoms
P_FDR = 0.6178
P_FDR = 0.2966
P_FDR = 0.1056
P_FDR = 0.2966
P_FDR = 0.2966
None
One or more
Neurological symptoms
P_FDR = 0.6178
P_FDR = 0.6331
P_FDR = 0.2966
P_FDR = 0.6607
P_FDR = 0.9422
None
One or more
Anosmia/dysgeusia symptoms
P_FDR = 0.6439
P_FDR = 0.6439
P_FDR = 0.3979
P_FDR = 0.3979
P_FDR = 0.6331
None
One or more
Gastrointestinal symptoms

Figure S5. IL2-AIS scores are correlated with post-acute sequelae symptoms in severe and critical COVID-19 patients.

A. Time-adjusted IL2-AIS scores for patients with or without post-acute sequelae of COVID-19 (PASC) symptoms, shown for the five cell types in the INCOV cohort. Each dot represents a patient. Patients are stratified by their maximum COVID-19 severity (colours), with mild patients shown on the top row, and severe/critical patients shown on the bottom row. The *P* values were calculated using two-sided Mann–Whitney *U* test and corrected for multiple testing using the Benjamini–Hochberg procedure. **B**. Time-adjusted IL2-AIS scores for severe/critical patients with or without specific categories of PASC symptoms. Each dot represents a patient. Colours represent the maximum severity of patients. The *P* values were calculated using two-sided Mann–Whitney *U* test and corrected for multiple testing using the Benjamini–Hochberg procedure.

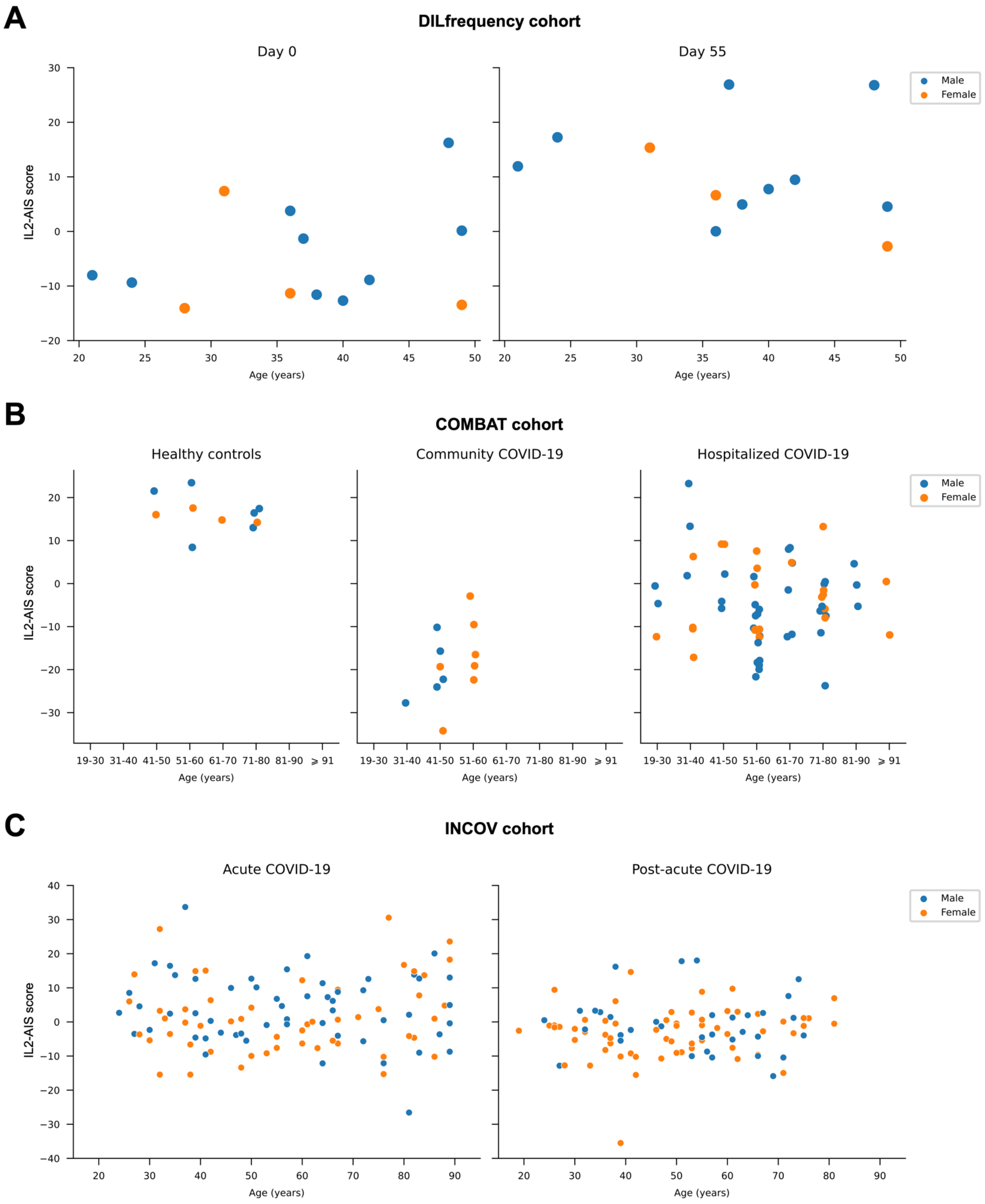
A
DILfrequency cohort
Day 0
Day 55
IL2-AIS score
Age (years)
Male
Female
B
COMBAT cohort
Healthy controls
Community COVID-19
Hospitalized COVID-19
19-30 31-40 41-50 51-60 61-70 71-80 81-90 ⩾ 91
C
INCOV cohort
Acute COVID-19
Post-acute COVID-19

Figure S6. The IL2-AIS score is not associated with age or sex.

A-**C**. Correlation between IL2-AIS scores, age, and sex in the DILfrequency (**A**), COMBAT cohort (**B**), and INCOV (**C**) cohorts. Each dot represents a participant, with colours representing the sex. In the INCOV cohort, IL2-AIS scores were calculated separately from samples collected in the acute (defined as the earliest sample taken 0-14 days post COVID-19 symptoms) or post-acute (defined as the earliest sample taken 29-84 days post COVID-19 symptoms) phases of the disease.

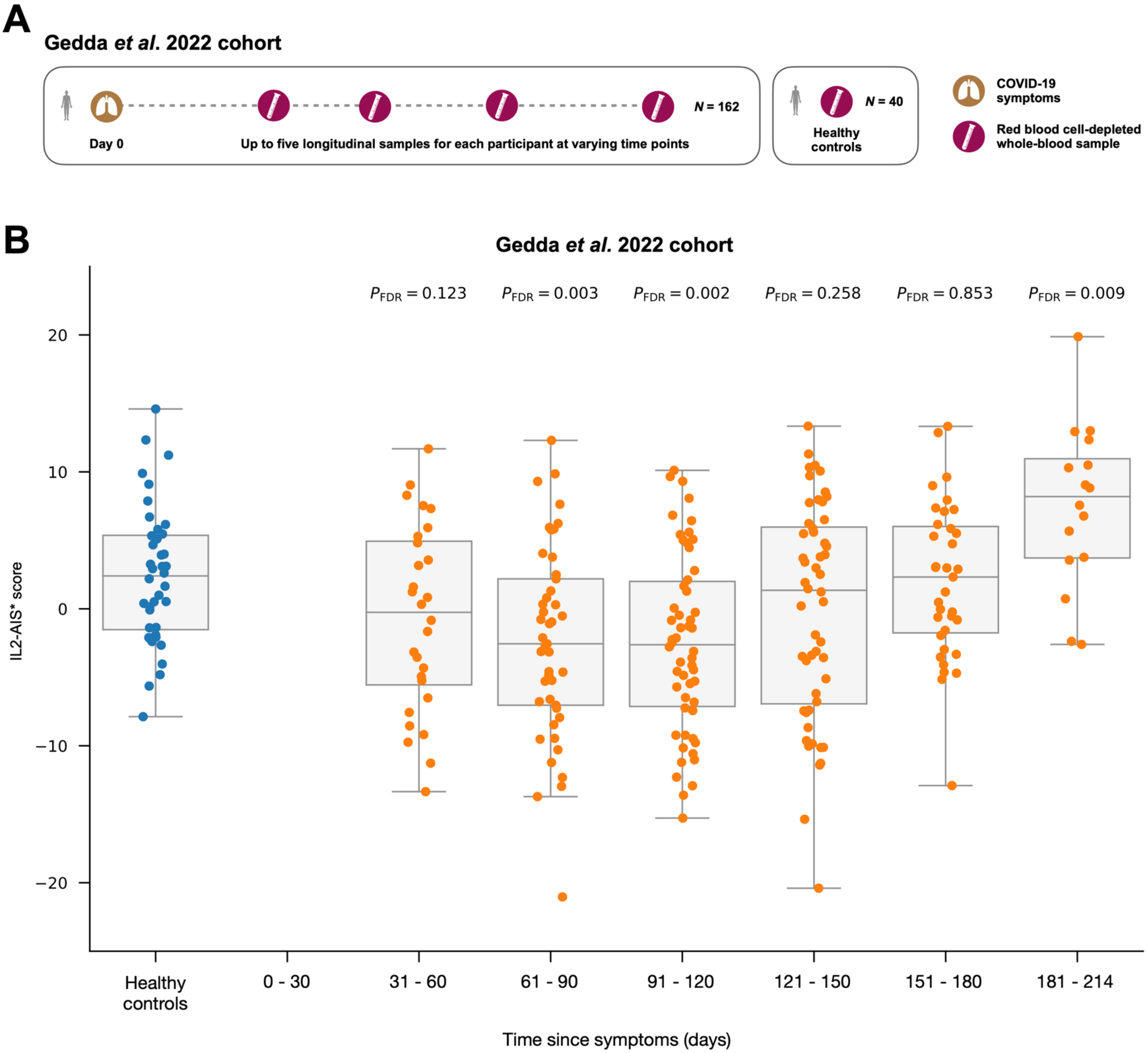

Figure S7. Replicating a subset of IL2-AIS using the NanoString nCounter transcriptomics platform.

A. Overview of the Gedda *et al*. 2022 cohort [7]. **B**. Distribution of the IL2-AIS* scores from 139 convalescent COVID-19 patients and 40 healthy control participants in the Gedda *et al*. 2022 cohort. The IL2-AIS* scores were calculated using a subset of 16 IL2-AIS constituent genes profiled using the NanoString nCounter Human Host Response panel: *MYC*, *CXCR1*, *OAS1*, *TNFSF10*, *FASLG*, *CCR10*, *STAT1*, *CX3CR1*, *CD40LG*, *IL32*, *EOMES*, *CCR1*, *OSM*, *SLC2A3*, *CXCR4*, and *CCL5*. Each dot represents a whole-blood sample from a participant visit. Each participant has up to five longitudinal samples. Each box ranges from the first quartile (Q1) to the third quartile (Q3), with a central line indicating the median. *P* values are calculated by comparing each group of COVID-19 patients with healthy controls using two-sided Mann–Whitney U test followed by FDR adjustment.

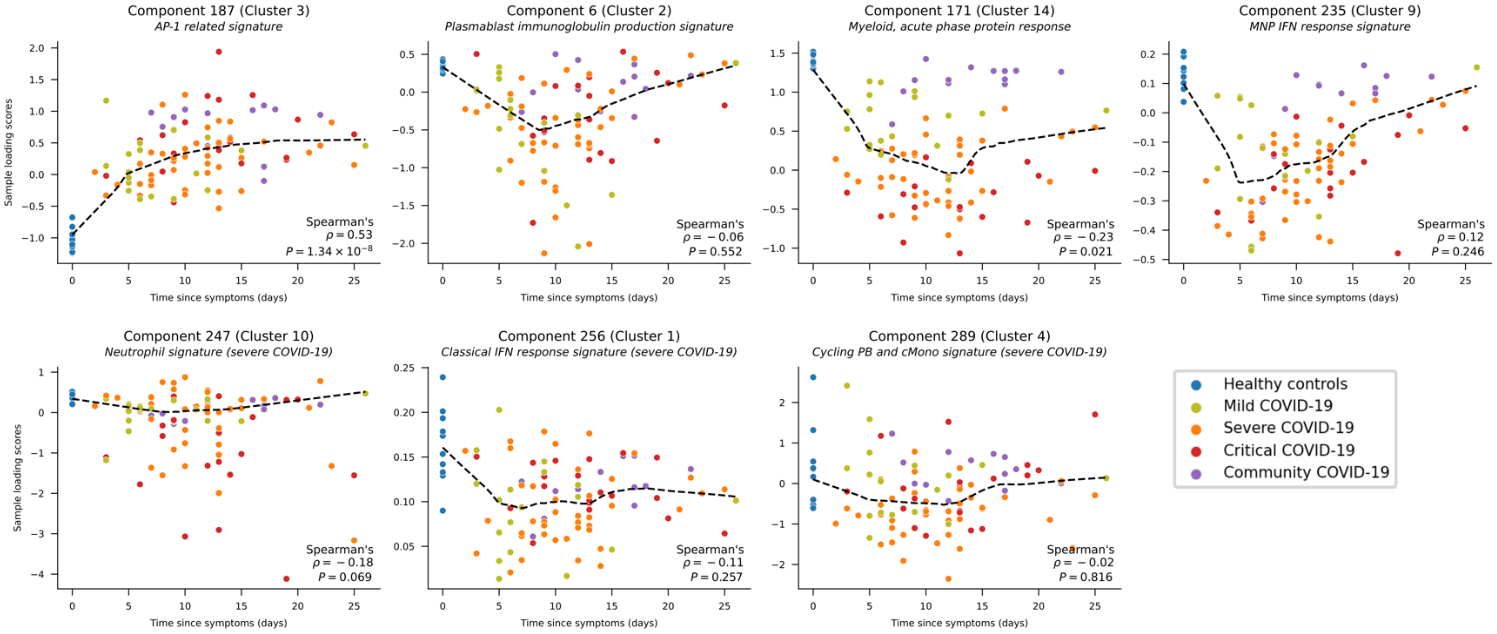

Figure S8. Temporal patterns of selected gene expression components in the COMBAT dataset.

The sample loading scores of seven gene expression components identified in the COMBAT dataset as being correlated with COVID-19 infection. Descriptions of each component are shown in italics. For each component, the direction of changes carries no predefined meaning and needs to be interpreted with respect to specific genes. Each dot represents a participant. Dashed black lines represent locally weighted scatterplot smoothing (LOWESS) curves.
